# Cigarette Smoking and Alcohol Consumption among Chinese Older Adults: Do Living Arrangements Matter?

**DOI:** 10.3390/ijerph120302411

**Published:** 2015-02-23

**Authors:** Jiaan Zhang, Liyun Wu

**Affiliations:** 1 Department of Social Work, The Chinese University of Hong Kong, Shatin, NT, Hong Kong, China; 2 The Ethelyn R. Strong School of Social Work, Norfolk State University, 700 Park Avenue, Norfolk, VA 23504, USA; E-Mail: lwu@nsu.edu

**Keywords:** smoking, drinking, living arrangements, old adults, China

## Abstract

This study used five waves of the Chinese Longitudinal Healthy Longevity Survey to examine the relationship between living arrangements, smoking, and drinking among older adults in China from 1998–2008. We found that living arrangements had strong implications for cigarette smoking and alcohol consumption among the elderly. First, the likelihood of smoking was lower among older men living with children, and older women living either with a spouse, or with both a spouse and children; and the likelihood of drinking was lower among both older men, and women living with both a spouse and children, compared with those living alone. Second, among dual consumers (*i.e.*, being a drinker and a smoker), the amount of alcohol consumption was lower among male dual consumers living with children, while the number of cigarettes smoked was higher among female dual consumers living with others, compared with those living alone. Third, among non-smoking drinkers, the alcohol consumption was lower among non-smoking male drinkers in all types of co-residential arrangements (*i.e.*, living with a spouse, living with children, living with both a spouse and children, or living with others), and non-smoking female drinkers living with others, compared with those living alone. Results highlighted the importance of living arrangements to cigarette smoking and alcohol consumption among Chinese elderly. Co-residential arrangements provided constraints on Chinese older adults’ health-risk behaviors, and had differential effects for men and women.

## 1. Introduction

Health-risk behaviors such as cigarette smoking and alcohol consumption are high-priority public health concerns as they are associated with a variety of detrimental health outcomes. Smoking has been linked to the cause of preventable and premature death [1,2]. Alcohol consumption is associated with an increased risk of cardiovascular diseases, raised blood pressure [3], and diseases of the liver [4]. In this regard, identifying and understanding risk and protective factors associated with smoking and alcohol consumption could help design possible prevention and intervention approaches to cut down on risks or counteract risks.

A considerable amount of research has focused on identifying factors that influence health behaviors. Some research shows that individual level characteristics such as age, gender, marital status, income, and educational attainment have been identified as having significant influence on engaging in health-risk behaviors [5,6]. Others suggest moving beyond the individual level in explaining health behaviors [7,8,9,10]. Of particular interest are family or household settings. An individual’s living arrangement provides social control that, as shown in a growing body of evidence, is associated with a healthier lifestyle and a lower incidence of health-risk behaviors [11,12,13,14,15,16,17,18,19,20].

Umberson, in her seminal work on social control of health behavior, argues that social control of health behavior can operate through both direct and indirect pathways [18,19]: Direct social control refers to actions such as persuasions, requests, threats, punishments, or rewards that urge individuals to give up health-risk behaviors or adopt health-enhancing behaviors. For example, spouses or children may tell an individual to avoid smoking or alcohol consumption and control the type of food available to an individual with a weight problem; or an individual might threaten to leave a spouse because of alcohol abuse. Indirect social control refers to avoiding health-risk behaviors or engaging in a healthier lifestyle through an internalized sense of responsibility or obligation to significant others. For example, the responsibility toward a child or a spouse leads individuals to control their own health-risk behaviors.

Previous studies find that co-residential arrangements constrain risky health behaviors. Marriage provides a well-defined social role that regulates and controls the individual’s behaviors; and the married traditionally are more likely to avoid risky behavior than the non-married [18,21,22]. Living with other non-spouse adults could be an alternative means to experience social control coming from marriage. Those non-married who do not live alone, like the married, are less likely to be involved in risky behavior [23].

The association between living arrangements and health behaviors may vary by gender due to gender differences in health behaviors and expectations, and obligations in family roles. Women are more likely than men to be responsive to health concerns and are less likely to engage in a number of risky health behaviors due to traditional gender role socialization [24,25,26]. In general, men smoke more than women [27]; Studies also consistently find that men drink more than women in different societies and cultures [28]. The gender differential in health-risk behaviors may further be reinforced by cultural norms. For instance, women’s smoking and drinking behaviors have traditionally been considered socially improper in China [29,30]. Moreover, women are more likely than men to take responsibility for providing care to others in the household [31,32]. Driven by this, women are more likely to monitor their own health status and influence others’ health behaviors more often than men [19]. There is evidence that living with a spouse is more beneficial for men’s health behaviors than for women’s because wives are more likely to exert health-related social control over their husbands than *vice versa* [19,20].

Although research on living arrangements and health behaviors is compelling, relatively little work has been conducted in older populations. To the best of our knowledge, most relevant studies have mainly focused on adolescents, and young and middle-aged adults [21,32,33,34]. It is still not clear whether the association between living arrangements and health behaviors found in these groups could be extended to older adults. Much less is known about health behaviors among older people in China, a country with a vast number of older adults. Although studies indicate that there are variations across different cultural groups in determinants of health behaviors [35,36], existing studies about elderly people’s decisions to engage in health behaviors have largely been conducted in Western countries. Our knowledge of what factors influence health behavior in other cultural contexts is still largely inadequate. This study fills these gaps. Using data collected from older adults in China, we aim to answer two questions: First, are different types of living arrangements (living alone, living with a spouse, living with children, living with both a spouse and children, and living with others) associated with health-risk behaviors, namely smoking and alcohol consumption, among the Chinese older adults? Second, are there gender differences in the association of living arrangements and health-risk behaviors?

This study focuses on contemporary China, a country experiencing rapid aging of its population. People aged 60 or older already comprised 14.4% of the total Chinese population in 2013 [37]. China also is the world’s largest tobacco manufacturing and consuming country with approximately 250 million smokers [38], and has experienced a dramatic increase in alcohol consumption since the late 1970s [39]. In addition, one of the most significant and unique features of Chinese social structure is its emphasis on the family [40]. Individuals form strong emotional bonds and close relationships among family members. Having multiple generations living under one roof is the ideal and the most common living arrangement for Chinese older adults, which is strongly influenced by traditional social norms [41] and promoted by housing shortages and government policies [42].

Based on the literature, we accordingly hypothesize that living arrangements will be significantly associated with older people’s health-risk behaviors, namely smoking and alcohol consumption. Among the different living arrangements being investigated, we expect that Chinese older adults living alone will be more likely to be involved in health-risk behaviors because they have less social control to constrain them, compared with those living in co-residential arrangements (*i.e.*, living with a spouse, living with children, living with both a spouse and children, or living with others). Intergenerational living arrangements are expected to show constraints on health-risk behaviors because of the availability of multiple sources of social control and fulfillment of a culturally ideal living arrangement in China.

We also hypothesize that men and women differ in the association of living arrangements and health-risk behaviors. Based on study findings [19,20], we particularly expect that having a spouse in the household will provide more restraints on men’s health-risk behaviors than on women’s.

## 2. Methods

### 2.1. Data and Sample

The data for this study were drawn from all five available waves—1998, 2000, 2002, 2005 and 2008 waves—of the Chinese Longitudinal Healthy Longevity Survey (CLHLS). The CLHLS is a longitudinal survey of older adults in China that began in 1998 and was administrated by Duke University. Using stratified probability sampling, the CLHLS randomly selected half of the counties and cities in 22 of the 31 provinces of China that cover 85% of the total population in China and interviewed all centenarians who voluntarily agreed to participate in the survey in the selected counties and cities [43]. For each centenarian, one octogenarian (aged 80–89) and one nonagenarian (aged 90–99) who lived nearby (in the same village, street, town, county or city) and met specific age and sex criteria were randomly selected and interviewed [43]. The CLHLS provides considerable information on demographics, socioeconomic status, health status, health and lifestyle, and living arrangements. The survey had an overall response rate of 88%, and the data have been assessed to be of high-quality [43]. Details of the survey sampling design can be found elsewhere [43,44,45].

The initial sample size was 9093 respondents in 1998, with 8636 elderly living in the community and 457 living in institutions. Respondents were subsequently followed in two- to three-year intervals. To ensure sufficient sample size in subsequent waves, new cases were added at each subsequent wave to replace those who died or were lost to follow-up within survey intervals. Of the original respondents in the 1998 wave, 52.51% were retained in 2000, 28.46% were retained in 2002, 11.22% were retained in 2005, and 3.86% were retained in 2008. Of the refresher sample included in 2000 for the first time, 57.81% were retained in 2002, 25.20% in 2005, and 9.45% in 2008. Similarly, 57.10% of the 2002 refresher sample were retained in 2005, and 33.43% in 2008. Of the 2005 refresher sample, less than half (43.94%) were retained in 2008. Lastly, a total number of 8837 new respondents were introduced to the survey in 2008. Appendix Table A1 shows the high attrition rates for each wave. By 2008, the CLHLS had experienced approximately 96.14% sample loss from its initial 1998 respondents, 90.55% sample loss from its 2000 refresher cohort, 66.57% sample loss from its 2002 refresher cohort, and 56.06% sample loss from its 2005 refresher cohort.

We pooled the five waves of data and organized them into a person-year structure. A respondent can appear in the sample from one to five times. On average, the respondents in our sample were observed 1.6 times across five time periods. We restricted the analytic sample to non-institutional respondents aged 60 and older. Missing data of study variables were modest (the highest was 9.05% for self-rated health), we used listwise deletion methods, which yielded 59,373 individuals including 25,851 males and 33,522 females for analysis.

### 2.2. Research Design

This study used a pooled cross-sectional and explanatory correlational study design. Even though the CLHLS is longitudinal, we did not apply the standard analytic strategy such as fixed effects model to investigate the impact of changes in living arrangements on changes in health-risk behaviors. There are three reasons for not applying longitudinal analysis. First, the attrition rates for the CLHLS were too high (see Appendix Table A1). Although longitudinal data are well known for tracing the dynamics of behaviors, and controlling for unobserved fixed characteristics, they are also disadvantageous due to attrition and its subsequent consequence of having biased estimates [46]. Second, there was little variation in the key variables across waves. As shown in Appendix Table A2, Table A3 and Table A4, as many as 76% of respondents did not change their living arrangements in two waves; 88% of respondents had no changes in smoking status; and 83% had no changes in drinking status. Third, the pooled cross-sectional data can increase sample size and allow us to investigate the research questions for a period lasting 10 years. Because the repeated measure for the subject can be correlated, we conducted the analysis by clustering observations within a subject across waves. Prior studies also demonstrate the feasibility of using pooled cross-sectional data to obtain robust estimates [47,48].

### 2.3. Measures

#### 2.3.1. Dependent Variables

The dependent variables included two types of behavior: smoking and alcohol consumption. We first dichotomously coded variables of smoking and alcohol consumption. If respondents answered “yes” to the question “Do you smoke at the present time?” they were classified as current smokers (=1). Similarly, if respondents answered “yes” to the question “Do you drink alcohol at the present time?” they were classified as current drinkers (=1).

Second, we created continuous variables representing the number of cigarettes smoked and the amount of alcohol consumption for current smokers and current drinkers, respectively. Cigarette smoking was measured by the total number of cigarettes smoked on a typical day for current smokers. Alcohol consumption was measured by the average amount of alcohol drunk per day for current drinkers, using the Chinese unit of measurement “*liang*”. (Note: 1 *liang* is equivalent to 1.7637 ounces or 50 grams.)

#### 2.3.2. Key Independent Variable

The key independent variable was “living arrangements”. The dataset included information on every person living in the household and their relationship to the elderly respondent. Based on these questions, a mutually exclusive five-category variable of living arrangements was created: (a) living alone; (b) living with a spouse (without children but may have others); (c) living with children (without the spouse but may have others); (d) living with both a spouse and children (may have others); and (e) living with others only (living with some persons other than children and spouse). They were converted into a set of dummy variables in the multivariate analysis, with living alone serving as reference group.

#### 2.3.3. Control Variables

We included two sets of control variables in the analyses: socio-demographic characteristics and health conditions. Socio-demographic characteristics included age, gender, ethnicity, rural/urban residence, education, and primary lifetime occupation. We coded age as three levels based on 10-year age groups (young-old = 60–69, old-old = 70–79, oldest-old = 80+) in order to look for potential differences in the effects of living arrangements on health behaviors, according to different aging stages: young-old, old-old, and oldest-old. Gender was dummy coded with female = 1 and male = 0. Ethnicity was treated as a dichotomous variable with 0 representing majority *Han* Chinese, and 1 indicating ethnic minority people (*Hui* Chinese, *Zhuang* Chinese, *Yao* Chinese, *Korean* Chinese, *Man* Chinese, *Mongol* Chinese, or others). Residents of rural areas were coded 1 and urban = 0. Following previous studies on Chinese older adults [48,49,50], we dichotomized education as having some formal education (= 1) or no formal education (= 0) because the majority of older people in China are illiterate. Primary lifetime occupation was measured as a 4-category variable: 1 = professionals or managerial personnel, 2 = non-agricultural workers, 3 = agricultural workers, and 4 = never formally employed including those who did not work outside the home.

Two indicators of health conditions were included: self-rated health, and cognitive function. Self-rated health was assessed using a 5-point Likert scale ranging from 1 = very bad to 5 = very good. Cognitive function was measured by a Chinese version of the Mini-Mental State Examination (MMSE), which has been reported to be a valid measure of cognitive functioning among Chinese older adults [51,52]. The MMSE measure had a Chronbach’s alpha of 0.98 in our sample. The scores of the MMSE ranged from 0–30, with higher scores indicating better cognitive function. Following Chinese MMSE cut points, respondents scored below 18 were regarded as cognitively impaired [52].

### 2.4. Data Analysis

The first step of analysis examined distributional differences for all the variables used in the analysis over the 1998, 2000, 2002, 2005, and 2008 waves. Second, logistic regression models were used to test whether living arrangements were associated with smoking and drinking behaviors. Lastly, Ordinary Least Squares (OLS) regression models were used to examine whether living arrangements were associated with number of cigarettes smoked and amount of alcohol consumed for smokers and drinkers, respectively. All models were controlled for socio-demographic characteristics (age, gender, ethnicity, rural/urban residence, education, and primary lifetime occupation), health conditions (self-rated health and cognitive function), and survey years, and adjusted for clustering of observations within a subject over time.

Because the use of categorical variables in regression analysis is often avoided due to confusion concerning interpretation, we adopted the commonly used strategies–dummy coding [53]. We compared other groups of the predictor variable with one specific group of that predictor variable. For example, regarding our key independent variable, the living arrangements, we compared living with a spouse, living with children, living with both a spouse and children, and living with others to the reference category living alone. When interpreting multivariate logistic regression results, all of the estimated odds ratios for the different categories of the living arrangements variable were calculated in relation to those who were living alone.

Among smokers and drinkers, we ran separate OLS regression models using three subsamples based on their smoking and drinking status. Smokers and drinkers are not a homogeneous group. Their reasons for smoking and drinking, their preference for smoking over drinking, or *vice versa*, are individualized [54]. The more information we can gather about smokers and drinkers, the more we can design services to meet their needs. For this purpose, we divide the total sample into three distinct subsamples: (1) dual consumers (*i.e.*, smokers who drink, drinkers who smoke) [55]; (2) non-drinking smokers; and (3) non-smoking drinkers. Out of the total sample, 5210 respondents both smoked and drank, 6741 were non-drinking smokers, and 8144 were non-smoking drinkers. The strength of using separate OLS regression models is that it allowed us to capture the heterogeneity within the sample. As discussed earlier, because smokers and drinkers were self-selected to retain certain behaviors, selection bias has been an inherent challenge to conduct analysis using observational data. By conducting analysis separately for three subgroups, we are able to stratify the sample and increase the validity.

The second research question, gender differences in the association of living arrangements and health-risk behaviors, was addressed in two steps. First, the interaction effects of gender and living arrangements on smoking and drinking were tested. All the interaction terms between gender and each type of living arrangements were found to be statistically significant (see Appendix Table A5), which indicated that the effects of living arrangements on smoking and drinking vary by gender. Then, we examined the association separately for males and females. These gender-stratified analyses allowed us to investigate the heterogeneous results by gender.

All analyses were performed by Stata 12.0 [56].

## 3. Results

### 3.1. Percentages of Smoking and Drinking by Living Arrangements over Time

The characteristics of the sample at each survey wave were summarized in Table 1. The most common living arrangement among respondents at each wave was living with children, with 69.68% of the sample in 1998, 64.59% in 2000, 52.04% in 2002, 51.09% in 2005, and 48.79% in 2008; and the percentages were observed monotonically decreasing in the past decade. The proportion of smokers was between 17.24% (in 2000) and 20.13% (in 2005) across five waves. The proportion of drinkers was also low across five waves: between 18.09% (in 2008) and 24.28% (in 1998). Among smokers, the highest average number of cigarettes smoked per day was found in 2008 (10.24 cigarettes) while smokers in 2000 smoked the lowest average number of cigarettes per day (8.44 cigarettes). Among those drinkers, the average amount of alcohol consumed per day was between 2.53 *liang* (4.46 ounces) in 1998 and 2.91 *liang* (5.13 ounces) in 2005.

Table 1 also presents the distributions of the socio-demographic characteristics and health conditions across waves. Results showed that respondents, on average, were oldest-old adults (mean age 85+ across waves), female, *Han* Chinese, with no formal schooling, and living in rural areas. In terms of their primary lifetime occupation, more than half of the sample worked as agricultural workers, and the percentages were increasing in the past decade (54.7% in 1998 and 66.8% in 2008). In comparison, the proportion of elderly people who were never formally employed decreased over time (21.12% in 1998 and 11.72% in 2008). In terms of health conditions, about one fifth of the sample was identified as cognitively impaired, and less than 20% respondents rated their health as very bad or bad.

**Figure 1:**
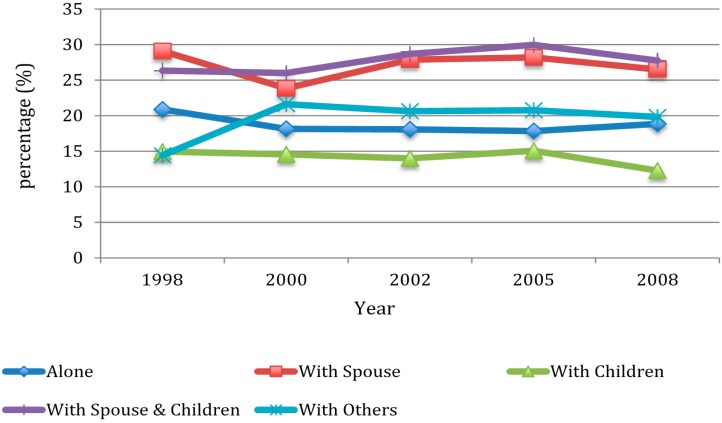
Percentage of smoking by wave and by living arrangement.

Figure 1 shows the proportion of smoking by living arrangement over 1998, 2000, 2002, 2005, and 2008 waves. In 1998, the highest proportion of smokers occurred among older adults living with a spouse (29.09%) while the lowest proportion of smokers occurred among those living with others (14.43%). The smoking patterns were quite similar over 2000, 2002, 2005, and 2008 waves with highest smoking rates among older adults living with both a spouse and children (26.01% in 2000, 28.69% in 2002, 29.96% in 2005, and 27.76% in 2008) and lowest rates among those living with children (14.56% in 2000, 14.00% in 2002, 15.07% in 2005, and 12.28% in 2008).

**Figure 2:**
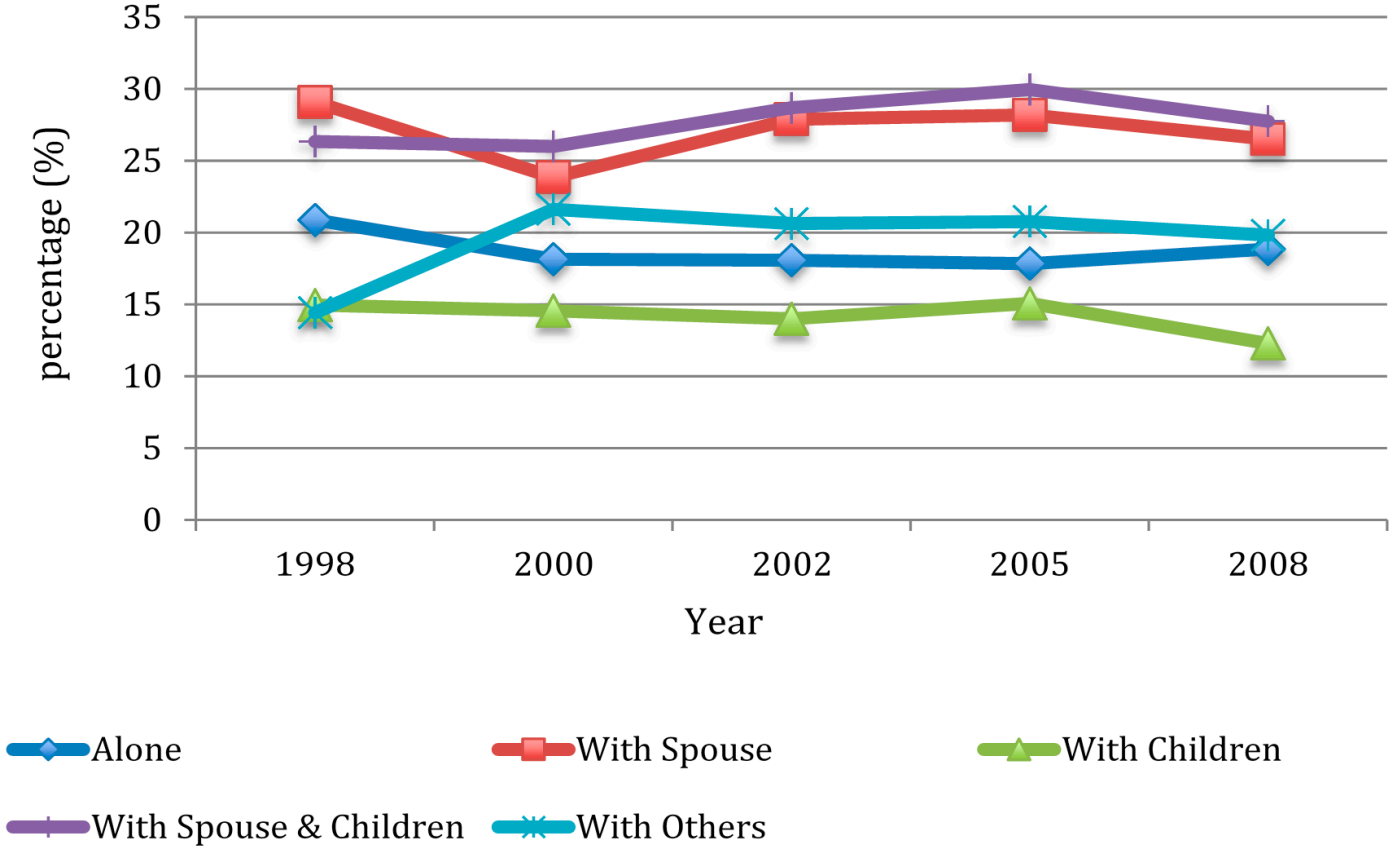
Percentage of drinking by wave and by living arrangement.

As shown in Figure 2, the percentage of drinkers was highest among those living with a spouse in 1998, 2002, and 2008; while the highest percentage of drinkers was found among those living with others in 2000, and among those living with both a spouse and children in 2005. Drinking rates were lowest among those living with others in 1998, and among those living alone in 2000. For 2002, 2005, and 2008, drinking rates were consistently low among those living with children.

**Figure 3:**
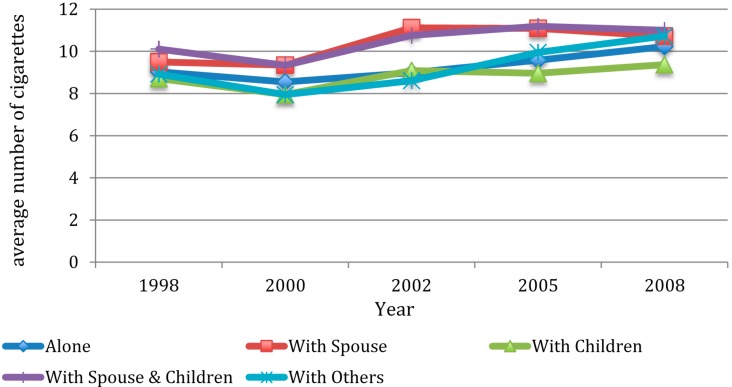
Average number of cigarettes smoked per day among current smokers by wave and by living arrangement.

**Figure 4:**
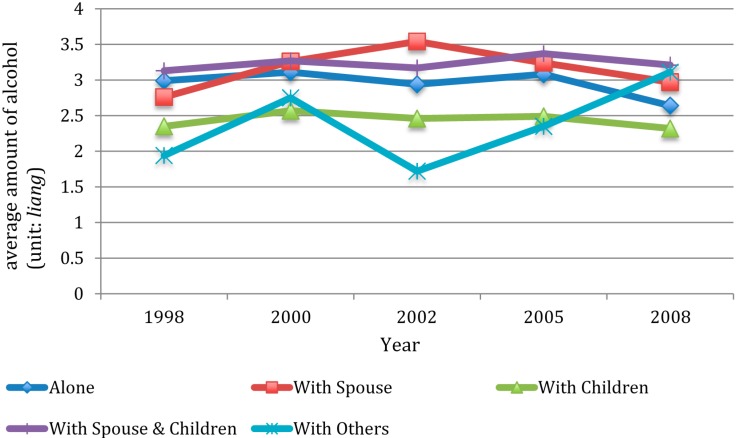
Average amount of alcohol consumed per day among current drinkers by wave and by living arrangement.

Figure 3 and Figure 4 showed average amount of smoking and drinking among current smokers and drinkers, respectively. As shown in Figure 3, the average number of cigarettes smoked by smokers in all types of living arrangements increased between 1998 and 2008, with those living with children having the lowest increasing rate. As shown in Figure 4, the average amount of alcohol consumed among those living with a spouse, with both a spouse and children, and with others increased between 1998 and 2008, whereas the drinking amount among those living alone, and living with children decreased between 1998 and 2008.

**Table 1:** Characteristics of the Sample.

	1998 (*n =* 7931)		2000 (*n =* 9461)		2002 (*n =* 13,957)		2005 (*n =* 13,909)		2008 (*n =* 14,115)	
	%	Mean (Std Dev)	%	Mean (Std Dev)	%	Mean (Std Dev)	%	Mean (Std Dev)	%	Mean (Std Dev)
Female	58.66		57.53		56.33		55.99		55.10	
Age		92.28 (7.70)		91.51 (7.58)		86.23 (11.80)		86.05 (11.73)		86.94 (11.70)
Age 60–69	0		0		11.64		12.01		11.32	
Age 70–79	1.29		0.29		22.31		22.95		19.54	
Age 80+	98.71		99.71		66.05		65.04		69.14	
Rural (*vs.* Urban)	63.85		39.58		55.01		56.14		60.16	
Minority (*vs.* Han Chinese)	7.66		6.50		5.79		6.18		6.62	
Some education (*vs.* None)	33.96		36.64		39.41		40.24		40.37	
Primary Lifetime Occupation										
Professionals or managerial personnel	8.12		8.41		9.71		9.38		8.14	
Non-agricultural workers	16.05		18.20		15.56		15.19		13.34	
Agricultural workers	54.70		52.31		58.56		61.08		66.80	
Never formally employed	21.12		21.08		16.17		14.35		11.72	
Cognitively impaired (*vs.* non-impaired)	20.80		19.06		16.93		17.49		18.06	
Self-rated health										
Very bad	0.56		0.92		1.38		1.62		1.42	
Bad	8.29		10.99		15.29		14.81		14.33	
So-so	33.90		31.68		35.50		33.92		34.19	
Good	44.35		40.75		37.17		38.73		38.41	
Very good	12.99		15.66		10.66		10.92		11.65	
Living Arrangements										
Alone	10.93		13.39		14.54		14.26		16.22	
With spouse	8.71		11.16		18.33		20.09		22.39	
With children	69.68		64.59		52.04		51.09		48.79	
With spouse & children	8.23		8.90		13.94		13.06		11.05	
With others	2.45		1.96		1.15		1.50		1.55	
Smoking	17.75		17.24		19.28		20.13		18.36	
Drinking	24.28		20.60		21.12		20.86		18.09	
# cigarettes smoked per day among current smokers		9.04 (6.54)		8.44 (6.19)		9.96 (7.12)		10.09 (7.21)		10.24 (7.47)
# alcohol consumed per day among current drinkers (unit: *liang*)		2.53 (2.34)		2.80 (2.79)		2.89 (2.74)		2.91 (2.89)		2.72 (2.39)

### 3.2. Effects of Living Arrangements on Smoking and Drinking Behaviors

This section presented results only from the gender-stratified analyses. The full sample regression models were not reported (see Appendix Table A5) due to the presence of the significant interactions between gender and each type of living arrangements.

Table 2 presents the results of logistic regression analyses to estimate the association between living arrangements and two dichotomous outcomes: the likelihood of smoking and drinking. Models 1 & 2 predicted the likelihood of smoking *vs.* non-smoking by gender. For males (model 1), the results showed the protective effect of living with children. Older males who lived with children were about 16% less likely to smoke than those who lived alone (OR = 0.84; 95% CI = 0.76–0.93). For older females (model 2), living with children only had no effect on smoking, while living with a spouse, or living with both a spouse and children, were both associated with significantly lower odds of smoking by 22% than for older females who lived alone.

Models 3 and 4, Table 2, predicted the likelihood of being a drinker *vs*. a non-drinker by gender. For both male and female, living with both a spouse and children put constraints on drinking behavior. Older men who lived with both a spouse and children had approximately 13% lower odds of drinking compared with those who lived alone (OR = 0.87; 95% CI = 0.78–0.97) (see Model 3). Older women who lived with both a spouse and children were associated with significantly lower odds of drinking (22%) than older women who lived alone (OR = 0.78; 95% CI = 0.64–0.97) (see Model 4).

As for the impact of socio-demographic characteristics and health conditions, young-old and old-old adults were more likely to smoke than their oldest-old counterparts. Young-old and old-old men were also more likely to drink than oldest-old men, while young-old women were less likely to drink than oldest-old women. Older people who were professionals or had managerial jobs were less likely to smoke than older people who never were formally employed, while agricultural male workers and non-agricultural female workers were more likely to smoke than their never formally employed counterparts. Older people with cognitive impairment were less likely to smoke. Older men with cognitive impairment were also less likely to drink. Meanwhile, good or very good self-rated health predicted greater likelihood of smoking and drinking for both genders.

**Table 2:** Multivariate logistics regressions predicting smoking and drinking among Chinese older adults: CLHLS, 1998–2008.

	Smoking (*vs.* Non-Smoking)		Drinking (*vs.* Non-Drinking)	
	Model 1	Model 2	Model 3	Model 4
	Male (*n =* 25,851)	Female (*n =* 33,522)	Male (*n =* 25,851)	Female (*n =* 33,522)
	OR (95% CI)	OR (95% CI)	OR (95% CI)	OR (95% CI)
Living arrangement (reference: Alone)				
With Spouse	0.94 (0.85–1.05)	0.78 * (0.64–0.96)	1.03 (0.93–0.14)	0.88 (0.73–1.05)
With Children	0.84 ** (0.76–0.93)	0.92 (0.80–1.05)	0.93 (0.84–1.02)	1.05 (0.94–1.17)
With Spouse & Children	0.95 (0.85–1.07)	0.78 * (0.62–0.98)	0.87 * (0.78–0.97)	0.78 * (0.64–0.97)
With Others	1.08 (0.85–1.37)	0.97 (0.66–1.43)	1.22 (0.95–1.55)	0.94 (0.69–1.27)
Age (reference: Age 80+)				
Age 60–69	2.41 *** (2.16–2.68)	1.46 *** (1.20–1.79)	1.66 *** (1.50–1.85)	0.82 * (0.68–0.98)
Age 70–79	1.78 *** (1.63–1.94)	1.34*** (1.15–1.57)	1.32 *** (1.21–1.44)	0.90 (0.78–1.03)
Minority (*vs.* Han Chinese)	0.91 (0.78–1.05)	0.66 ** (0.52–0.84)	1.32 *** (1.14–1.52)	0.91 (0.76–1.08)
Rural (*vs.* Urban)	1.44 *** (1.34–1.55)	0.87 * (0.78–0.96)	1.27 *** (1.18–1.36)	1.07 (0.98–1.16)
Some education (*vs.* None)	0.96 (0.89–1.04)	1.06 (0.91–1.24)	1.06 (0.98–1.14)	0.91 (0.80–1.03)
Jobs (reference: Never formally employed)				
Professionals or managerial personnel	0.77 ** (0.64–0.91)	0.54 ** (0.37–0.77)	0.85 (0.72–1.00)	0.86 (0.63–1.16)
Non-agricultural workers	0.99 (0.84–1.16)	1.24 * (1.02–1.50)	0.95 (0.81–1.11)	0.97 (0.83–1.14)
Agricultural workers	1.18 * (1.02–1.37)	0.98 (0.86–1.13)	1.14 (0.98–1.31)	1.16 (1.05–1.28)
Cognitively impaired (*vs.* Non-impaired)	0.75 *** (0.67–0.83)	0.85 ** (0.76–0.96)	0.74 *** (0.67–0.82)	0.98 (0.89–1.06)
Self-rated health (reference: Very bad)				
Bad	1.29 (0.97–1.72)	1.28 (0.82–2.01)	1.37 (0.99–1.88)	1.56 (1.03–2.37)
So-so	1.57 ** (1.18–2.08)	1.38 (0.89–2.15)	2.02 *** (1.47–2.75)	2.05 ** (1.36–3.07)
Good	1.78 *** (1.35–2.36)	1.58 * (1.02–2.47)	2.49 *** (1.83–3.41)	2.53 *** (1.68–3.80)
Very good	1.61 ** (1.21–2.15)	1.74 * (1.09–2.75)	2.63 *** (1.91–3.61)	2.57 *** (1.69–3.90)
Survey year (reference: 1998)				
2000	1.01 (0.92–1.09)	0.97 (0.86–1.11)	0.91 * (0.83–0.98)	0.76 *** (0.69–0.84)
2002	0.87 ** (0.79–0.95)	0.89 (0.78–1.03)	0.89 * (0.81–0.97)	0.70 *** (0.64–0.78)
2005	0.92 (0.84–1.01)	0.91 (0.78–1.04)	0.88 * (0.81–0.97)	0.64 *** (0.58–0.71)
2008	0.80 *** (0.73–0.88)	0.73 *** (0.63–0.85)	0.75 *** (0.68–0.82)	0.45 (0.40–0.51)

Note: OR = Odds Ratio; CI = Confidence Interval; * *p* < 0.05, ** *p* < 0.01, *** *p* < 0.001.

### 3.3. Effects of Living Arrangements on Amounts of Smoking and Drinking

Table 3 and Table 4 present the results from OLS linear regression models that examined the effects of living arrangements on amounts of smoking and drinking by using three sub-samples of smokers and drinkers.

#### 3.3.1. Dual Consumers Sample

Table 3 presents the results of the effects of living arrangements on number of cigarettes smoked per day and amount of alcohol consumed per day, among dual consumers. For male dual consumers, living arrangements had no significant effects on number of cigarettes smoked per day but put constraints on the amount of alcohol consumed. Specifically, living with children decreased the amount of alcohol consumed by 0.36 units (about 0.63 ounces) on a daily basis, compared with male dual consumers who lived alone. For female dual consumers, living arrangements were not significantly associated with the amount of alcohol consumed, but living arrangements were associated with an increase in the number of cigarettes smoked. Specifically, living with others increased the number of cigarettes smoked by almost six cigarettes per day, compared with those women who were dual consumers and lived alone.

**Table 3:** OLS regression estimates of number cigarettes smoked and amount of alcohol consumed among dual consumers (*n =* 5210): CLHLS, 1998–2008 ^a^.

	Number of Cigarettes Smoked		Amount of Alcohol Consumed	
	Model 1	Model 2	Model 3	Model 4
	Male (*n =* 4412)	Female (*n =* 798)	Male (*n =* 4412)	Female (*n =* 798)
	Coefficient (95% CI)	Coefficient (95% CI)	Coefficient (95% CI)	Coefficient (95% CI)
Living arrangement (reference: Alone)				
With Spouse	0.15 (−0.62–0.92)	0.42 (−1.52–2.35)	0.04 (−0.27–0.36)	−0.17 (−0.86–0.51)
With Children	−0.42 (−1.13–0.29)	−0.05 (−1.27–1.16)	−0.36 * (−0.64–−0.07)	−0.45 (−1.02–0.11)
With Spouse & Children	−0.37 (−1.22–0.48)	1.67 (−1.68–5.03)	−0.01 (−0.36–0.33)	0.01 (−0.98–1.00)
With Others	−0.30 (−1.84–1.24)	5.86 * (0.71–11.01)	−0.40 (−0.91–0.11)	1.05 (−1.89–3.99)

Note: * *p* < 0.05; ^a^ Models include the following variables: age, ethnic minority, rural residence, education, jobs, cognitive impairment, self-rated health, and survey year. Coefficient and confidence intervals of these variables are not displayed.

#### 3.3.2. Non-Drinking Smokers Sample

Models 1 and 2 in Table 4 present the results of the effects of living arrangements on number of cigarettes smoked per day for non-drinking smokers. As shown in Model 1 and Model 2 (Table 4), living arrangements were not significantly associated with number of cigarettes smoked for non-drinking smokers.

**Table 4:** OLS regression estimates of number cigarettes smoked and amount of alcohol consumed for non-drinking smokers (*n =* 6741) and non-smoking drinkers (*n =* 8144), respectively: CLHLS, 1998–2008 ^b^.

	Number of Cigarettes Smoked (Non-Drinking Smokers)		Amount of Alcohol Consumed (Non-Smoking Drinkers)	
	Model 1	Model 2	Model 3	Model 4
	Male (*n =* 4896)	Female (*n =* 1845)	Male (*n =* 4422)	Female (*n =* 3722)
	Coefficient (95% CI)	Coefficient (95% CI)	Coefficient(95% CI)	Coefficient (95% CI)
Living arrangement (reference: Alone)				
With Spouse	−0.23 (−0.99–0.53)	0.27 (−1.11–1.65)	−0.40 * (−0.78–−0.03)	−0.06 (−0.42–0.30)
With Children	−0.15 (−0.84–0.55)	0.31 (−0.54–1.18)	−0.73 *** (−1.05–−0.41)	−0.07 (−0.28–0.13)
With Spouse & Children	−0.11 (−0.89–0.67)	0.083 (−0.82–2.48)	−0.54 ** (−0.94–−0.14)	−0.08 (−0.47–0.31)
With Others	−0.82 (−2.57–0.94)	−0.61 (−2.46–1.24)	−0.76 * (−1.37–−0.15)	−0.56 ** (−0.91–−0.22)

Note: * *p* < 0.05, ** *p* < 0.01, *** *p* < 0.001; ^b^ Models include the following variables: age, ethnic minority, rural residence, education, jobs, cognitive impairment, self-rated health, and survey year. Coefficient and confidence intervals of these variables are not displayed.

#### 3.3.3. Non-Smoking Drinkers Sample

Models 3 and 4 in Table 4 present the results of the effects of living arrangements on amounts of alcohol consumed per day for non-smoking drinkers. We found that living arrangements had significant effects on the amount of alcohol consumed for non-smoking drinkers. For male non-smoking drinkers (Model 3), living in multiple-member households constrained the amount of alcohol consumption, compared with living alone. Specifically, living with a spouse, living with children, living with both a spouse and children, and living with others decreased the amount of alcohol consumed per day by about 0.71 ounces, 1.29 ounces, 0.95 ounces, and 1.34 ounces, respectively. For female non-smoking drinkers (Model 4), living with others appeared to be a protective factor that decreased the amount of alcohol consumed by about 0.99 ounces per day, compared with persons living alone.

## 4. Discussion

Previous studies of adolescents and adults indicate that living arrangements are associated with health behaviors [21,22,23,24]. This study extended existing knowledge to the group of older adults. Household setting is the most important immediate environment for older adults. In this study, we examined the relationship between living arrangements and health-risk behaviors (smoking and alcohol consumption) among community-dwelling older adults using a longitudinal, nationally representative dataset of China. The main results of the present study supported our first hypothesis that living arrangements of Chinese older adults have significant association with their smoking and drinking behaviors, and our second hypothesis that the influence of living arrangements on health-risk behaviors is gendered.

Consistent with our hypothesis that co-residential arrangements would constrain health-risk behaviors, living with children, living with a spouse, and living with both a spouse and children were found to decrease the likelihood of older adults’ smoking and drinking behaviors, compared with living alone; all types of co-residential arrangements in our sample (with a spouse, with children, with both a spouse and children, and with others) were associated with a lower amount of alcohol consumption for male drinkers, compared with living alone. Female drinkers who lived with others consumed less alcohol than those who lived alone. This finding somewhat echoes Buckingham’s notion that “living alone means living at risk” [57]. We suspect that social control and psychological experience such as loneliness may be among the mechanisms linking living alone and engaging in health-risk behaviors. The social control of the health behavior argument [18,19,21] implies that individuals constrain their risky behaviors as a reaction to the external influence. Living alone makes those influences unavailable, leading to fewer constraints on negative behaviors, while co-residential arrangements provide the “control agents” [18,19,21] (children and spouse, and other co-resident people) to constrain or regulate health-risk behaviors. Moreover, living alone is associated with a higher likelihood of feeling lonely [58]. Research showed that older people living alone suffered more from loneliness than those living with someone else [59]. Lonely individuals were more likely to be smokers out of the desire to reach out to other people, especially in regions in which smoking is socially acceptable [60], or to engage in alcohol consumption in order to reduce stress and cope with feelings of isolation and unworthiness [58,61].

Our results also supported the hypothesis that intergenerational living arrangements provide constraints on Chinese older adults’ health-risk behaviors. We found that living with children was associated with significantly lower odds of smoking for older men, and lower amounts of alcohol consumption for male dual consumers (*i.e.*, being a drinker and a smoker); older women’s smoking behaviors were also constrained in a household setting with a spouse and children. Living with both a spouse and children decreased the likelihood of alcohol consumption for both genders. The situation of older parents living with children is traditionally preferred and reinforced by social norms in China. Although there have been dramatic changes in the Chinese society in recent decades, the majority of the elderly in China still live with their adult children [40]. Intergenerational support within families is currently the major source of old age security and care in Chinese society [62]. Filial responsibilities of children refer to the belief and expectation that adult children will provide support for their parents and play a crucial role in their life [63]. One possible way of children influencing their older parents’ health behaviors may be through direct social control from taking actions such as distribution of health information (e.g*.*, smoking and alcohol consumption are hazardous to health), persuasion, requests, and encouragement that their older parents not adopt health-risk behaviors.

Previous research suggests that spouses generally are listed as the most influential social control agent [16,19]. Wives, in particular, are more successful in influencing husbands’ negative health behaviors than husbands are in influencing wives to change [64]. Our results on spouses’ effect on health-risk behaviors, however, contradict our hypothesis that wives are more effective in restraining husbands’ health-risk behaviors. We found that living with a spouse decreased the likelihood of smoking only for older women. In other words, husbands provided significant restraint on wives’ smoking behaviors, while wives did not have this kind of control on husbands’ smoking behaviors. One possible explanation of this inconsistency with Western literature is that the dynamics between the couple varies across different cultural and social contexts. In China, the status is traditionally unequal between men and women in a marital relationship [65]. Influenced by Confucianism, the husband–wife relationship was strictly held to be subordinate to the father–son relationship, which means the wife should be loyal and obedient to her husband as the son is obliged to be loyal and obedient to his father. We could imagine that older women who are strictly bound by this traditional social norm will have less control over their husbands’ health-risk behaviors than their younger Western counterparts.

One interesting finding is that female dual consumers (*i.e.*, being a drinker and a smoker) who lived with others smoked more cigarettes than those who lived alone. This finding somewhat contradicts the social control of health behavior argument. This finding can be explained in several ways. One possible explanation is that the effectiveness of social control of health behavior may depend on the relationship with the social control agent. Positive relationships support engagement in better health practices [64,66]. If individuals experience frustration and threats to personal autonomy during the interaction with the control agent, they will have a greater tendency to ignore the social control agent and less likelihood to adopt the desired behavior [13,16,17,23]. Some studies, on the other hand, did find that negative interactions with the social control agents (e.g., criticism, demands), nevertheless, induced positive behavior change [66,67,68]. This body of work suggests that people may tolerate negative tactics of social control of health behaviors exercised by family members such as spouse and children [66,67,68,69]. However, the likelihood of positive behavioral reaction to negative tactics of social control may be very low in our study, considering that our measure of living with others refers to living with some persons other than children and spouse. In addition, it could also be argued that the observed association might be due to the choice of living arrangement: smokers may tend to live with other smokers rather than with non-smokers. Our dataset lacks information about the nature and the quality of relationships with co-residents or co-residents’ health-risk behaviors. Future research is needed to examine these mechanisms.

These findings should be interpreted with the following limitations in mind. First, the study used a pooled cross-sectional design and therefore cannot claim any causal relationships between living arrangements and health-risk behavior outcomes (smoking and drinking). Second, our study did not examine the dynamics of living arrangements and health risk behaviors over time due to data limitation. Future research might want to examine whether changing living arrangements are associated with changes in health risk behaviors over time. Third, we did not distinguish between different types of alcohol beverages (e.g., beer, grape wine, rice wine, and liquor) when we measured drinking behaviors. This is largely due to incomplete information of types of alcohol beverages provided by the dataset. Fourth, this sample of older adults (average age of each wave was 85+) may represent a unique cohort that had experienced extreme hardships in their lifetime, including war, famine, and major political upheaval. It is unknown whether our results would apply to other cohorts of older Chinese. Despite these limitations, this study uses a large nationally representative dataset of older adults from China, and contributes to the literature by examining an understudied issue of the association between living arrangements and Chinese older adults’ health-risk behaviors.

## 5. Conclusions

This study indicates that living arrangements of Chinese older adults are important predictors of their smoking and drinking behaviors. Findings suggest that living alone generally is a risk factor for engaging in smoking and alcohol consumption because of lack of social control. As more and more older adults around the world live alone [70], there is a growing concern about the well-being of this group. One implication from this study is the need for efforts to increase health-related social control to regulate and exert a positive influence on the health behaviors of older adults. Policies should be promoted that encourage children to have frequent contact with and visit their older parents. Services should be developed that offer regular visits by health workers to older parents and monitor their health behaviors.

## Appendix

**Table A1:** Data structure & attrition for the Chinese elderly living in the community.

Variable list	Stay in 1998	Stay in 2000	Attrition Rate (%)	Stay 2002	Attrition Rate (%)	Stay 2005	Attrition Rate (%)	Stay 2008	Attrition Rate (%)
Baseline cohort 1998	8636	4535	47.48	2458	71.54	969	88.78	333	96.14
Refresher sample in 2000	–	5885	–	3402	42.19	1483	74.80	556	90.55
Refresher Sample in 2002	–	–	–	9470	–	5407	42.90	3166	66.57
Refresher sample in 2005	–	–	–	–	–	7358	–	3233	56.06
Refresher sample in 2008	–	–	–	–	–	–	–	8837	–

**Table A2:** Variation in living arrangement for five waves.

Categories	N	No Changes in Living Arrangement N (%)	Having Changes in Living Arrangement N (%)
Living arrangement is available only in one wave	23,609	–	–
Living arrangement in two waves	9470	7194 (75.97%)	2276 (24.03%)
Living arrangement in three waves	5582	3302 (59.15%)	2280 (40.85%)
Living arrangement in four waves	1192	658 (55.20%)	534 (44.80%)
Living arrangement in five waves	332	169 (50.90%)	163 (49.10%)

**Table A3:** Variation in smoking for five waves.

Categories	N	No Changes in Smoking N (%)	Having Changes in Smoking N (%)
Smoking is available only in one wave	23,612	–	–
Smoking in two waves	9487	8379 (88.32%)	1108 (11.68%)
Smoking in three waves	5563	4587 (82.46%)	976 (17.54%)
Smoking in four waves	1189	960 (80.74%)	229 (19.26%)
Smoking in five waves	332	275 (77.41%)	75 (22.59%)

**Table A4:** Variation in drinking for five waves.

Categories	N	No Changes in Drinking N (%)	Having Changes in Drinking N (%)
Drinking is available only in one wave	23,612	–	–
Drinking in two waves	9492	7845 (82.65%)	1647 (17.35%)
Drinking in three waves	5560	4111 (73.94%)	1449 (26.06%)
Drinking in four waves	1189	800 (67.28%)	389 (32.72%)
Drinking in five waves	330	211 (63.94%)	119 (36.06%)

**Table A5:** Multivariate logistic regression predicting smoking and drinking among Chinese older adults: CLHLS, 1998–2008 pooled sample with and without interaction terms.

	Smoking (*vs.* Non-Smoking)		Drinking (*vs.* Non-Drinking)	
	Main Effect Model	Interaction Model	Main Effect Model	Interaction Model
	OR (95% CI)	OR (95% CI)	OR (95% CI)	OR (95% CI)
Living arrangement (Reference: Alone)				
With Spouse	0.91 (0.83–1.00)	2.23 *** (2.02–2.45)	1.02 (0.94–1.11)	2.04 *** (1.87–2.24)
With Children	0.87 *** (0.81–0.95)	1.95 *** (1.78–2.13)	0.99 (0.93–1.07)	1.70 *** (1.57–1.85)
With Spouse & Children	0.93 (0.84–1.02)	2.24 *** (2.02–2.48)	0.88 ** (0.80–0.97)	1.72 *** (1.56–1.90)
With Others	1.07 (0.88–1.31)	2.47 *** (1.97–3.11)	1.12 (0.93–1.35)	2.25 *** (1.78–2.85)
Female (*vs.* Male)	0.15 *** (0.14–0.16)		0.28 *** (0.26–0.29)	
Interaction Terms				
Female * w/spouse		0.11 *** (0.09–0.13)		0.18 *** (0.15–0.21)
Female * w/children		0.18 *** (0.17–0.20)		0.36 *** (0.33–0.38)
Female * w/spouse & children		0.11 *** (0.09–0.14)		0.19 *** (0.16–0.24)
Female * w/others		0.17 *** (0.11–0.26)		0.25 *** (0.17–0.36)
Age (reference: Age 80+)				
Age 60–69	2.12 *** (1.94–2.32)	2.07 *** (1.89–2.27)	1.36 *** (1.25–1.48)	1.38 *** (1.26–1.50)
Age 70–79	1.67 *** (1.55–1.80)	1.61 *** (1.49–1.73)	1.18 *** (1.10–1.27)	1.17 *** (1.09–1.26)
Minority (*vs.* Han)	0.84 ** (0.74–0.94)	0.83 ** (0.74–0.94)	1.13 * (1.02–1.26)	1.13 * (1.01–1.25)
Rural (*vs.* Urban)	1.23 *** (1.16–1.30)	1.26 *** (1.18–1.34)	1.19 *** (1.13–1.26)	1.21 *** (1.15–1.28)
Some education (*vs*. None)	0.98 (0.91–1.05)	1.14 *** (1.07–1.22)	1.01 (0.95–1.07)	1.13 *** (1.06–1.20)
Jobs (Reference: Never formally employed)				
Professionals	0.63 *** (0.55–0.72)	0.71 *** (0.62–0.81)	0.80 *** (0.71–0.90)	0.85 ** (0.76–0.96)
Non-agricultural workers	0.88 * (0.78–0.99)	1.01 (0.89–1.13)	0.87 * (0.79–0.96)	0.95 (0.86–1.05)
Agricultural workers	0.99 (0.90–1.10)	1.13 * (1.03–1.25)	1.06 (0.98–1.15)	1.16 *** (1.07–1.25)
Cognitively impaired (*vs.* Non-impaired)	0.80 *** (0.74–0.87)	0.78 *** (0.72–0.84)	0.90 ** (0.85–0.96)	0.88 *** (0.82–0.94)
Self-rated health (reference: Very bad)				
Bad	1.29 * (1.01–1.63)	1.27 * (1.01–1.61)	1.46** (1.14–1.88)	1.45 ** (1.13–1.86)
So-so	1.51 *** (1.19–1.91)	1.50 *** (1.19–1.89)	2.06 *** (1.61–2.64)	2.05 *** (1.60–2.62)
Good	1.72 *** (1.36–2.18)	1.71 *** (1.36–2.15)	2.55 *** (1.99–3.26)	2.53 *** (1.98–3.23)
Very good	1.63 *** (1.28–2.08)	1.64 *** (1.29–2.08)	2.65 *** (2.06–3.41)	2.65 *** (2.06–3.40)
Survey year (reference: 1998)				
2000	0.99 (0.93–1.07)	0.99 (0.93–1.07)	0.83 *** (0.78–0.89)	0.84 *** (0.78–0.89)
2002	0.87 *** (0.81–0.94)	0.89 ** (0.82–0.95)	0.78 *** (0.73–0.84)	0.80 *** (0.74–0.85)
2005	0.91 *** (0.84–0.98)	0.93 (0.86–1.00)	0.76 *** (0.71–0.82)	0.77 *** (0.72–0.83)
2008	0.78 *** (0.72–0.85)	0.80 *** (0.74–0.87)	0.61 *** (0.57–0.66)	0.62 *** (0.58–0.67)

Note: OR = Odds Ratio; CI = Confidence Interval; * *p <* 0.05, **** *p <* 0.01, **** p <* 0.001.
